# Differential molecular information of maurotoxin peptide recognizing IK_Ca _and Kv1.2 channels explored by computational simulation

**DOI:** 10.1186/1472-6807-11-3

**Published:** 2011-01-25

**Authors:** Hong Yi, Su Qiu, Yingliang Wu, Wenxin Li, Baoshan Wang

**Affiliations:** 1 State Key Laboratory of Virology, College of Life Sciences, Wuhan University, Wuhan, 430072, PR China; 2 College of Chemistry and Molecular Sciences, Wuhan University, Wuhan 430072, PR China

## Abstract

**Background:**

Scorpion toxins are invaluable tools for ion channel research and are potential drugs for human channelopathies. However, it is still an open task to determine the molecular basis underlying the diverse interactions between toxin peptides and ion channels. The inhibitory peptide Maurotoxin (MTX) recognized the distantly related IK_Ca _and Kv1.2 channel with approximately the same potency and using the same functional residues, their differential binding mechanism remain elusive. In this study, we applied computational methods to explore the differential binding modes of MTX to Kv1.2 and IK_Ca _channels, which would help to understand the diversity of channel-toxin interactions and accelerate the toxin-based drug design.

**Results:**

A reasonably stable MTX-IK_Ca _complex was obtained by combining various computational methods and by in-depth comparison with the previous model of the MTX-Kv1.2 complex. Similarly, MTX adopted the β-sheet structure as the interacting surface for binding both channels, with Lys23 occluding the pore. In contrast, the other critical residues Lys27, Lys30, and Tyr32 of MTX adopted distinct interactions when associating with the IK_Ca _channel. In addition, the residues Gln229, Ala230, Ala233, and Thr234 on the IK_Ca _channel turret formed polar and non-polar interactions with MTX, whereas the turret of Kv1.2 was almost not involved in recognizing MTX. In all, the pairs of interacting residues on MTX and the IK_Ca _channel of the bound complex indicated that electrostatic and Van der Waal interactions contributed equally to the formation of a stable MTX-IK_Ca _complex, in contrast to the MTX-Kv1.2 binding that is dominantly mediated by electrostatic forces.

**Conclusions:**

Despite sharing similar pharmacological profiles toward both IK_Ca _and Kv1.2 channels, MTX adopted totally diverging modes in the two association processes. All the molecular information unveiled here could not only offer a better understanding about the structural differences between the IK_Ca _and Kv1.2 channels, but also provide novel structural clews that will help in the designing of more selective molecular probes to discriminate between these two channels.

## Background

Scorpion venoms produce a large variety of peptide toxins that target ion channels [1-5]. Especially, the widespread use of scorpion-venom peptides acting on K^+^-channels as neuroscience tools and excellent ligand models has tremendously increased our knowledge in many fields, including exploration of the 3-dimensional structures and elucidation of the pharmacological characteristics of K^+ ^channels [4,6-8]. In addition, peptide toxins are increasingly recognized as valuable sources of new drugs for channelopathies [9,10]. Although natural toxins often lack sufficient efficacy and specificity toward an individual channel type, most peptide toxins adopt a cysteine-stabilized α/β scaffold; thus, they could serve as good candidates for further structure-based drug design [4,10]. However, crystal structures for many medically important potassium channels have not been determined, which makes the rational designing of K^+^-channel modulators difficult. Therefore, applying computational methods to model reasonably stable structures of channel-peptide toxin complexes could be a good alternative, which would greatly help to highlight the diversity of channel-toxin interactions and provide structural information for toxin-based drug design.

The intermediate-conductance calcium-activated potassium channels (IK_Ca_) act as positive modulators of cell proliferation by hyperpolarizing the cell membrane in T and B cells, fibroblasts, and vascular smooth muscle cells [11-13]. Furthermore, blocking of IK_Ca _channels has been shown to be a potential therapeutic strategy against autoimmune disorders involving these tissues [13-15]. However, almost all the peptidic and small molecular IK_Ca _blockers could not discriminate well between the IK_Ca _channel and other related Kv-family channels and, thus, lack the specificity needed for further drug development [12,13,15,16].

Maurotoxin (MTX), a peptide derived from the venom of the scorpion Scorpio maurus palmatus, is the most potent peptidic blocker of the IK_Ca _channel [17,18]. In addition, MTX could distinguish the IK_Ca _channel from the other calcium-activated channels and the Kv1-family channels, except for the voltage-gated Kv1.2 channel [17-21]. Interestingly, although the IK_Ca _channel is entirely different from the Kv1.2 channel in tissue contribution and physiological function [11,12,15,16], MTX shows very similar pharmacological profiles in recognizing these two channels with approximately the same potency and using the same functional residues [17,18,21]. In this study, we aimed to interpret the differential binding mechanisms of MTX with reference to the IK_Ca _and Kv1.2 channels, which would provide a deep insight into the topological differences of these two channels and offer important clues for designing inhibitors that are more selective toward the therapeutic IK_Ca _channel.

Combined computational methods were used to investigate the details of the interactions between MTX and the IK_Ca _channel; the structural details were further compared with the previous model of the MTX-Kv1.2 complex [22]. A stable structure of the MTX-IK_Ca _complex was obtained by using ligand docking, clustering analysis, and molecular dynamics simulation (MDS) methods. The validity of the final MTX-IK_Ca _complex was supported by good accordance between the computational alanine-scanning results and the experimental data. On comparison, although with similar pharmacological profiles, MTX adopted very different modes for associating with the IK_Ca _and the Kv1.2 channels. In both MTX-IK_Ca _and MTX-Kv1.2 complexes, MTX adopted the β-sheet domain as the interaction surface with the Lys23 occluding the pore. However, the other key residue, Tyr32, was positioned quite differently in these two complexes. Meanwhile, the turret region of the IK_Ca _channel played an important role in binding with MTX, which is different from the noninvolvement of the Kv1.2 turret during interaction with MTX. In addition, due to the different physicochemical profiles of the two channels, electrostatic and van der Waals (vdW) interactions made different contributions to the free energies of binding in MTX-Kv1.2 and MTX-IK_Ca _complexes. All these structural and energetic discrepancies constitute the key determinants responsible for the binding specificity of MTX to the IK_Ca _and Kv1.2 channels, which could help design MTX derivatives that would discriminate between these two channels.

## Results and Discussion

### Different MTX binding modes towards IK_Ca _and Kv1.2 channels

**Figure 1:**
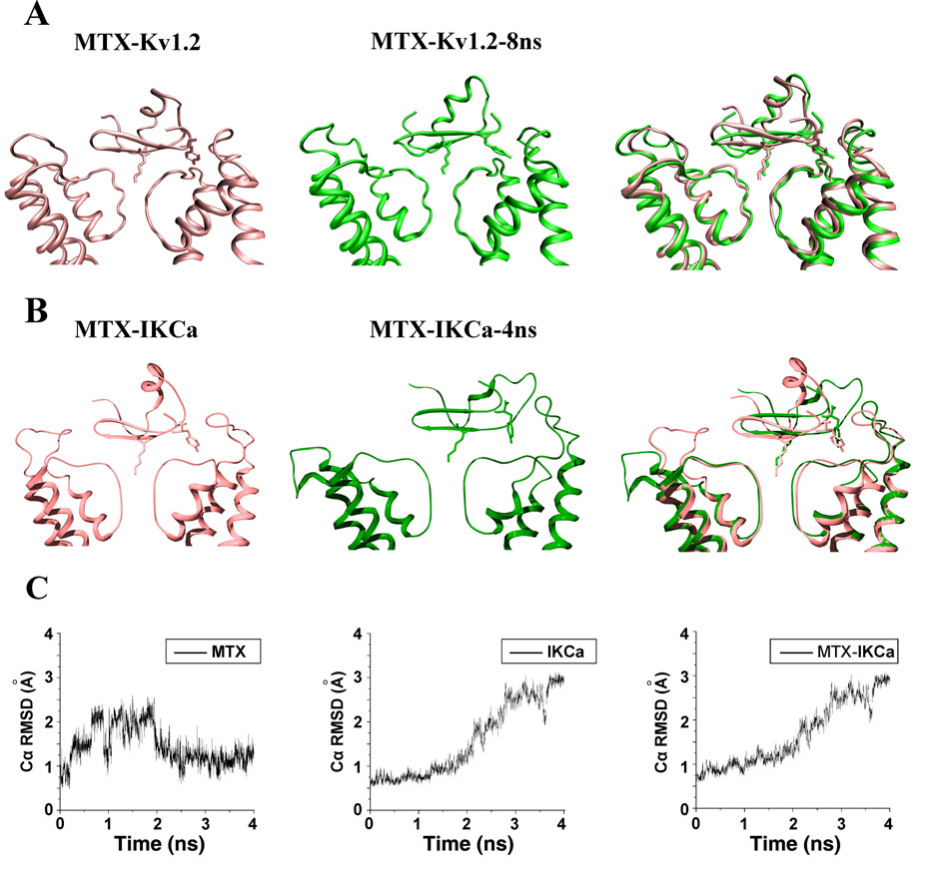
**The stability of an MTX-IK_Ca _candidate that resembles the MTX-Kv1.2 complex**. (A) The structural comparison of the MTX-Kv1.2 complex before and after 8-ns unrestrained MDS. (B) The structural comparison of the MTX-Kv1.2-like candidate for the MTX-IK_Ca _complex before and after 4-ns unrestrained MDS. Complexes before and after MDS are distinguished by colors. (C) The RMSd fluctuation of MTX (left), IK_Ca _(middle) and complex (right) of the MTX-Kv1.2-like candidate for the MTX-IK_Ca _complex in 4-ns MDS.

Given the similar blocking activities that MTX showed toward IK_Ca _and Kv1.2 channels [17,18], we first tested whether MTX recognized the IK_Ca _channel in the same binding mode as for the Kv1.2 channel. An MTX-IK_Ca _complex was modeled on the basis of the MTX-Kv1.2 complex structure [22] using the distance-restraint homologous modeling method and then subjected to unrestrained MDS to test its stability. Previously, MTX adopted the β-sheet domain as the interacting surface, with Lys23 occluding the pore of the Kv1.2 channel (Figure 1A, left panel). And the most important residue, Tyr32, of MTX kept sticking into the pocket formed by residues on the turret and pore region of the Kv1.2 channel during the 8-ns production run [22] (Figure 1A). Thus, the interaction between MTX and the Kv1.2 channel was beneficial for the stability of the turret region. On the contrary, in the MTX-IK_Ca _complex, in spite of starting from a similar conformation of sticking into the pocket formed by the turret and the pore region (Figure 1B, left panel), the Tyr32 was unwelcome in this position and gradually bent away from the "pocket" after 4-ns MDS (Figure 1B, middle panel). Therefore, the experimentally important residues, Lys23 and Tyr32, failed to form strong interactions with the channel, and MTX deviated from the central position (Figure 1B, right panel). As a result, the turret on the other side of the IK_Ca _channel failed to maintain stability and bent outward (see Figure 1C the RMSD of IKCa channel). Obviously, this complex structure could not explain the experiment results at all. Therefore, although MTX showed similar pharmacological profiles toward both Kv1.2 and IK_Ca _channels, it did not use the same binding mode in associating with these two channels.

### MTX-IK_Ca _complex from docking and MDS

We next applied a routine molecular docking and clustering analysis to screen plausible MTX-IK_Ca _complexes [22-25]. All the 35 nuclear magnetic resonance conformations of MTX with different side-chain positions were used in the ZDOCK program, and 35, 000 complexes were generated in total.

**Figure 2:**
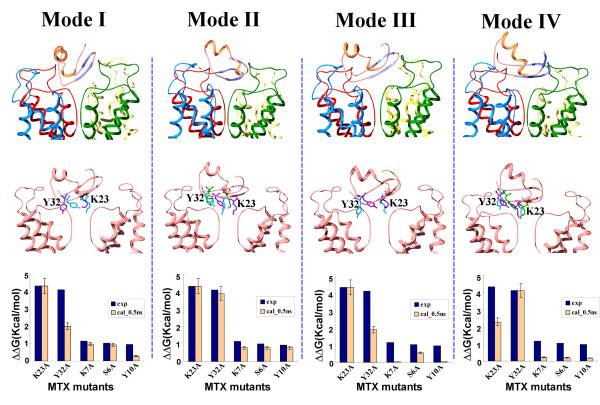
**Structural view of the four selected MTX-IK_Ca _channel-binding modes along with the comparison of the calculated and experimental effects**. Each row of figures is as follows: (top) the different spatial orientations of MTX in its complex with the IK_Ca _channel from four main binding modes; (middle) the representative positions of the Lys23 and Tyr32 side chains in four main binding modes distinguished by different colors; (bottom) the comparison of the calculated and experimental effects for the five alanine mutations of MTX on the binding affinity toward the IK_Ca _channel after 500-ps unrestrained MDS. The calculated results are normalized values of ΔΔ*G*_binding_, whereas the experimental results are obtained as *k_b_T *ln [IC_50_(mutant)/IC_50_(wt)].

**Figure 3:**
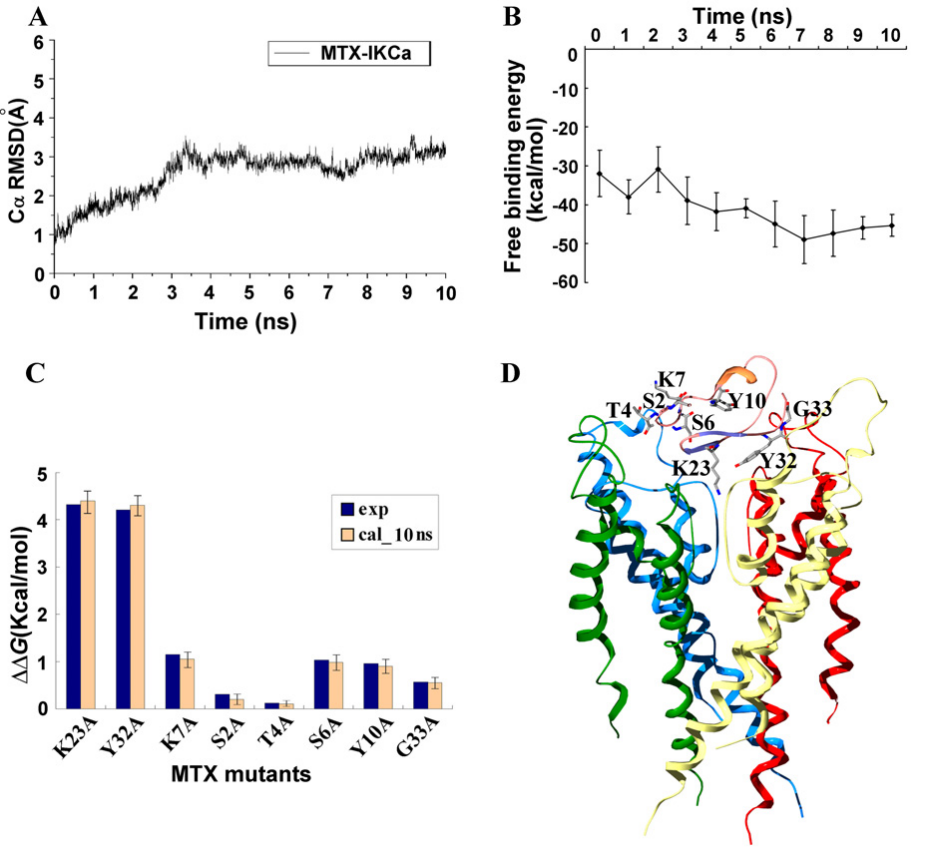
**The stability and validity of the final MTX-IK_Ca _complex**. (A) Root-mean-square deviations (Å) of the α-carbons in the final MTX-IK_Ca _complex from the starting complex during the 10-ns unrestrained MD simulations. (B) The free energy convergence during the 10-ns unrestrained MD simulations, trajectories were sampled every ns along the simulation time. (C) Calculated and experimental effects for 5 single-alanine mutations on the blocking activity of MTX toward the IK_Ca _channel after 10-ns MDS. The calculated results are normalized values of ΔΔ*G*_binding_, whereas the experimental results are obtained as *k_b_T *ln [IC_50_(mutant)/IC_50_(wt)]. (D) An overview of the final model of the MTX-IK_Ca _complex, with the labels of all the experimentally determined residues.

The 35, 000 MTX-IK_Ca _complexes fell into four main binding modes, according to the orientation of the MTX β-sheet domain (Figure 2, top panel). As indicated in our previous study, the starting position of the residue Tyr32 of MTX is essential for obtaining a final stable complex [22]. Thus, we mapped all possible orientations of the aromatic ring of Tyr32 in the four groups of complexes (Figure 2, middle panel), including the following: (1) lying slantways over the linker connecting S6 and the selectivity filter of the IK_Ca _channel; (2) hanging between two subunits of the channel; (3) plugging the pore of the channel along with Lys23; (4) hanging upright over the linker connecting S6 and the selectivity filter of the IK_Ca _channel. Next, each of the candidates in the four binding modes was subjected to energy minimization, followed by 500-ps unrestrained MDS, to introduce more flexibility and investigate the potential structural fluctuations. For better discrimination among the four binding modes, the alanine-scanning method in the MM-GBSA of Amber-8 was applied for comparison of the computational and experimental data of functional residues (Figure 2, bottom panel). Finally, an MTX-IK_Ca _model that best fit the experimental data was selected from the binding mode II. To make it more equilibrated and stable, the selected MTX-IK_Ca _model was subjected to a further 10-ns unrestrained MDS (Figure 3A). According to the major transitions during the MDs, we also evaluated the free energy convergence by sampling along the whole simulation (Figure 3B). The results showed that both the peptide and the channel were induced to fit in their nanosecond-scale diffusional encounter, and then guided to form a specific stable complex.

To check the confidence of our MTX-IK_Ca _model, the ΔΔ*G*_binding _of eight single mutations of MTX were calculated and compared with the experimental data [17,18]. An overall high degree of correlation was found between the calculations and the experiments involving mutational effects (Figure 3C). Replacing the Lys23 residue of MTX with alanine caused the most noticeable decrease of 4.4 kcal/mol in the calculated binding energy, which is well in accordance with the experimental data of 4.32 kcal/mol. Substitution of another important residue, Tyr32, with alanine significantly reduced the MTX affinity by over 1000 fold [18], and the calculated ΔΔ*G*_binding _value of 4.31 kcal/mol corresponded well with the experimental data of 4.21 kcal/mol. However, MTX affinity for IK_Ca _was decreased by less than 10 fold by the S2A, T4A, S6A, K7A, Y10A, and G33A mutants [18]. This is strongly supportive for the little change in binding energy when these residues were mutated to alanine; this is because Ser2, Thr4, Ser6, and Lys7 were located at the N-terminal of MTX, whereas Tyr10 was in the middle of the α-helix of MTX, all outside the interface of MTX.

Consistent with the findings in previous docking experiments of MTX onto IK_Ca _channel [26], in the final MTX-IK_Ca _complex, the peptide used its β-sheet as the interacting surface, with Lys23 as a structurally conserved pore-blocking residue (Figure 3D). This phenomenon was also observed when MTX associated with the Kv1.2 channel in both our study [22] and the previous docking results by Visan [18]. All these studies underline the key role of MTX β-sheet region in IK_Ca _and Kv1.2 channel recognition. Interestingly, such importance is strongly supported by the previous experimental data that when substituting the β-sheet region of MTX with that of another toxin, HsTX1, its activity toward the two channels almost disappeared [27].

Despite that MTX used the β-sheet to interact with both IKCa and Kv1.2 channels, when analyzing the conformation of other bioactive residues, the molecular information for the recognition of IK_Ca _by MTX showed several distinct features, compared to those for the Kv1.2 channel.

### Differential molecular information contained by Tyr32 and Lys7 in MTX

**Figure 4:**
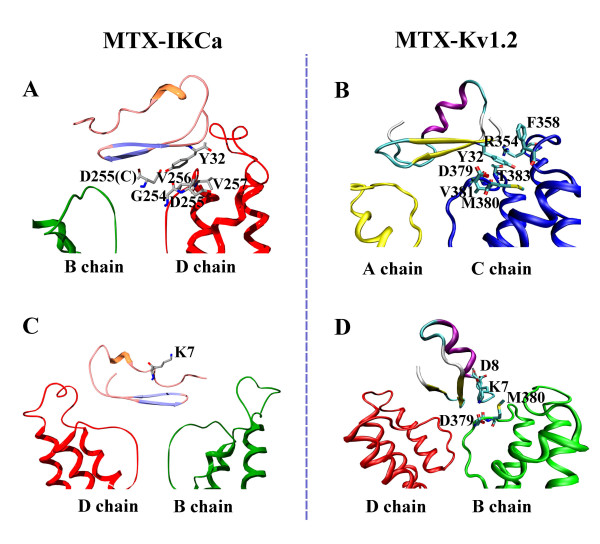
**The distinct recognition mechanisms of MTX toward the IK_Ca _and Kv.1.2 channels**. (A), (C) Interaction details of Tyr32 and Lys7 of MTX in the MTX-IK_Ca _complex within a contact distance of 5Å, respectively. (B), (D) Interaction details of Tyr32 and Lys7 of MTX in the MTX-Kv1.2 complex within a contact distance of 5Å, respectively.

The mutant-cycle experiment showed that two major functional residues, namely, Lys23 and Tyr32, in MTX block the IK_Ca _channel [17,18]. The key role of these residues can be shown by their structural conformation in the final MTX-IK_Ca _complex. Although the pore-blocking characteristic of Lys23 was common when MTX recognized both the IK_Ca _and the Kv1.2 channels, the conformation of Tyr32 of MTX differed greatly in each of these contexts (Figure 4A, B). As indicated in the second section, the residue Tyr32 is essential for MTX-associating potassium channels, and its position was proposed to be the main factor determining the stability of the complex structure after a long-term unrestrained production run. In the final MTX-IK_Ca _complex, the favorable position for the residue Tyr32 was lying on the linker connecting the selectivity filter and the S6 helix of the channel, forming strong polar and nonpolar interactions with Gly254 (D chain), Asp255 (C and D chains), Val256 (D chain), and Val257 (D chain) on the pore region of the channel (Figure 4A). In comparison, the Tyr32 in the MTX-Kv1.2 complex, however, differed significantly from that in the MTX-IK_Ca _complex by sticking into the pocket formed by Arg354 and F358 on the turret and Asp379, Met380, Val381, and Thr383 on the pore region of the Kv1.2 channel [22] (Figure 4B). Thus, the differential chemical environment for Tyr32 contributed greatly to the different binding modes of MTX when associating with the IK_Ca _and Kv1.2 channels.

In addition, rather than forming strong electrostatic interactions with the aspartic acid residues in the pore region of the channel, the side chain of the Lys7 of MTX pointed to an opposite orientation from the IK_Ca _channel, contacting no residue of the channel within a 5-Å distance (Figure 4C). This is different from the result of the mutant-cycle analysis that Lys7 is situated near the Asp239 of the IK_Ca _channel [17,18]. However, as the K7A mutation only affected the blocking activity of MTX by less than 10 fold [17,18], it is possible that the Lys7 just faced its alternative partner Asp239 in the interface reorganization process, but does not contact Asp239 directly in the final conformation. Such a position of the Lys7 while associating with the IK_Ca _channel differed significantly from that in the MTX-Kv1.2 complex, in which the Lys7 formed strong polar interactions with the Asp373 at the pore region of the channel [22] (Figure 4D). This distinctness was in consistence with the experimental data that the blocking activity of the MTX-K7A mutant decreased by about 100 fold in the case of the Kv1.2 channel, but decreased by only less than 10 fold in the case of the IK_Ca _channel [18].

### Differential molecular information of other residues in MTX

**Figure 5:**
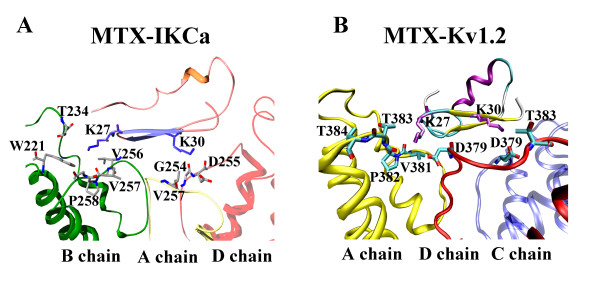
**The comparison for the pairs of MTX-IK_Ca _and MTX-Kv1.2 interacting residues in the bound complexes**. Lys27 and Lys30 of MTX are surrounded by residues from the IK_Ca _channel (A) and Kv1.2 channel (B) within a distance of 5Å, respectively.

Considering that the β-sheets of MTX constitute its main channel-interacting surfaces, two other residues Lys27 and Lys30 were also found to play outstanding roles in the recognition process (Figure 5A, B). In the model of the MTX-IK_Ca _complex, the Lys30 mainly interacted with Gly254 (D chain) and Val257 (A chain) at the pore region of the IK_Ca _channel (Figure 5A); in addition, it also formed some electrostatic interactions with the backbone of Asp255 (D chain). Another basic residue Lys27, however, did not electrostatically interact with any acidic residues of the channel, but rather stuck into a pocket formed mostly by nonpolar residues, including Thr234 and Trp221 on the turret; and Val256, Val257, and Pro258 on the linker connecting the S6 helix and the selectivity filter (Figure 5A). Such finding is completely in line with the previous docking result of MTX onto IK_Ca _channel [26], which highlighted the important contribution of Lys27 and Lys30 in the interaction, due to their proximity to Asp255, Val257 and Asp239 of the channel.

However, these interaction modes mediated through Lys27 and Lys30 of the MTX in recognizing the IK_Ca _channel differed obviously from those involving MTX and the Kv1.2 channel, in which both Lys27 and Lys30 formed strong polar interactions with the channel [22]. Within a contact distance of 5Å, the Lys27 and Lys30 of MTX, respectively, contacted closely with the conserved acidic residue, Asp379, in the pore region of the Kv1.2 channel and formed strong electrostatic interactions (Figure 5B).

The different functional roles for the Lys27 and Lys30 of MTX when recognizing the IK_Ca _and the Kv1.2 channels could be further illustrated by calculating their mutation effects. As indicated in Table 1, either changing Lys27 or Lys30 of MTX into alanine resulted in a big change in the MTX-IK_Ca _and MTX-Kv1.2 interactive energies. However, in the model of the MTX-IK_Ca _complex, the changes in the vdW energies and electrostatic energies almost synergistically contributed to the large values of the altered interactive energies when replacing Lys27 with alanine (Table 1), in accordance with the observation that the Lys27 of MTX mainly interacted with the nonpolar residues in IK_Ca _channel. In contrast, in the MTX-Kv1.2 complex, the increase in the interactive energies caused by mutating Lys27 and Lys30 were both dominantly constituted by the significant change in the electrostatic energies (Table 1), resulting from the interacting residue pairs Lys27-Asp373 and Lys30-Asp379 (Figure 5B).

**Table 1:** Effects of K27A and K30A mutants on interactive energies (Kcal/mol)

Energies	MTX-IK_Ca_			MTX-Kv1.2		
Mutants	*ΔE*_vdW_	*ΔE*_ele_	*ΔE*_inter_	*ΔE*_vdW_	*ΔE*_ele_	*ΔE*_inter_
WT	-78.65	-118.21	-196.86	-74.06	-1577.51	-1651.57
MTX_K27A	-74.31	-110.24	-184.55	-61.62	-1361.07	-1422.69
MTX_K30A	-73.51	-73.07	-146.58	-57.01	-1354.70	-1411.71

Interactive energies (kcal/mol) were calculated using the MM-GBSA program in Amber-8.

### Distinct channel vestibules constitute different recognition modes toward maurotoxin

The α-KTx family of K^+^-channel blockers has been proved to function as informative molecular probes for the structure-function analysis of K^+ ^channels. Although the IK_Ca _and Kv1.2 channels have distinct tissue distributions and biophysical features [11-16], both can be blocked by MTX with a similar pharmacology profile. Thus, identifying the differential determinants that are responsible for the MTX binding of the IK_Ca _and Kv1.2 channels could help discover the different topologies of a mechanistically interesting part of these two channels: the outer vestibule of the ion-conduction pore.

**Figure 6:**
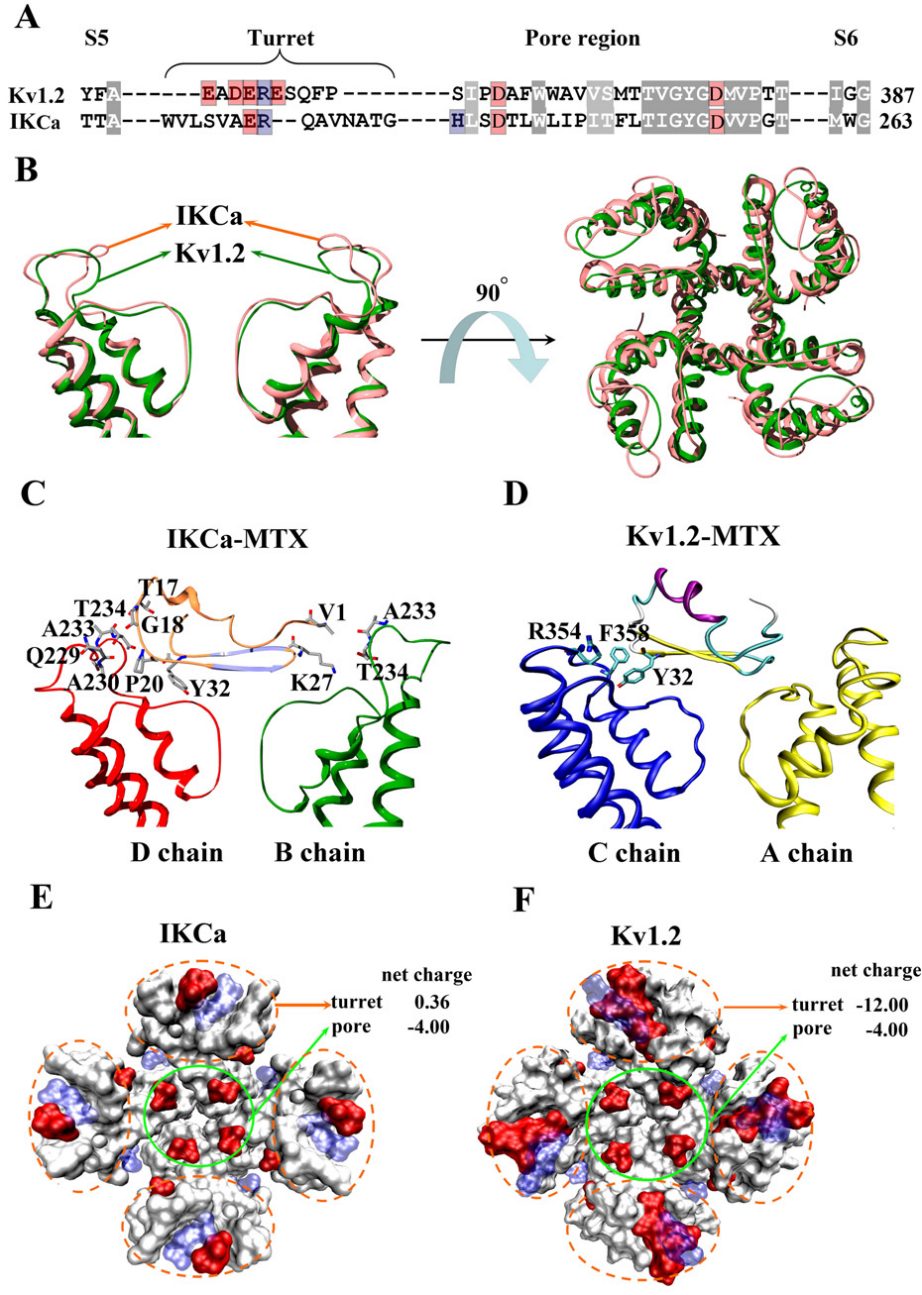
**The comparison for the sequence, conformation, and functional role of the outer vestibules of the IK_Ca _and Kv1.2 channels**. (A) Sequence alignment for the outer vestibule of the IK_Ca _and Kv1.2 channels. The conserved residues are grey-shaded, acidic residues red-shaded, basic residues blue-shaded. (B) Left and right are, respectively, the side and top views of the ribbon structures of the outer vestibules of the IK_Ca _and the Kv1.2 channels, respectively. The IK_Ca _channel is pink-colored, and the Kv1.2 channel is green-colored. For clarity, only two chains of the channels are shown in the side view. (C), (D) Functional roles for the turrets of the IK_Ca _and Kv1.2 channels, respectively, in associating with MTX, respectively. All the toxin-interacting residues within a contact distance of 5Å are labeled on the turret of the IK_Ca _and Kv1.2 channels. (E), (F) Stereoview displaying the molecular surfaces of the IK_Ca _and Kv1.2 channels, respectively. The molecular surfaces are colored according to their electrostatic potentials, with basic residues in blue, acidic residues in red, and neutral residues in white.

Sequence comparison showed that the IK_Ca _channel has a longer turret region than the Kv1.2 channel, and the sequence identity is rather low (Figure 6A). These features were further shown by the structural analysis of unbound IK_Ca _and Kv1.2 channel vestibules (Figure 6B), whereas the differential conformation of the channel turret suggested a different functional role for the channel vestibule during the toxin-recognition process. This hypothesis was supported by comparing our model of the IK_Ca_-MTX complex with the previous Kv.1.2-MTX model [22] (Figure 6C, D). We mapped all the toxin-interacting residues in the IK_Ca _channel turret (see Figure 6C and additional file 1: Figure S1) and found that the channel turret played an important role in stabilizing the final IK_Ca_-MTX complex. Four residues, Gln229, Ala230, Ala233, and Thr234 in the IK_Ca _turret formed polar and non-polar interactions with the corresponding residues of MTX. Interactions have been found between Gln229 of IK_Ca _and Gly18, Pro20 of MTX, Ala230 of IK_Ca _and Pro20 of MTX, Ala233 of IK_Ca _and Val1, Thr17 of MTX, Thr234 of IK_Ca _and Lys27, Tyr32, Gly33 of MTX, respectively. Thus, the important role of the IK_Ca _channel's turret in recognizing MTX is in sharp contrast with the almost nil involvement of the Kv1.2 channel turret, because only the Tyr32 of MTX was able to contact the Kv1.2 turret within the distance of 5Å (Figure 6D) and several mutations on the Kv1.2 channel turret hardly affected the binding affinity of MTX [18,22]. In addition, it is noticeable that the total net charge of the turret of the IK_Ca _channel is almost neutral, due to the average distribution of positive and negative residues in the turret (Figure 6A, E). This is very different compared with the Kv1.x family, all of which contained four extremely negative-charged turrets (Figure 6A, F). Actually, the IK_Ca _channel turret, with an important functional role and neutral charge, could well explain the equal contribution of electrostatic and vdW interactions in mediating the recognition process between the IK_Ca _channel and MTX (Table 2), and the highly negative-charged Kv1.2 turrets are responsible for the dominance of electrostatic recognition in the binding of MTX to the Kv1.2 channel (Table 2). All these data suggest that the diverse vestibules of the IK_Ca _and Kv1.2 channels, including the sequence length, sequence identity, vestibule conformation, and property of molecular surface jointly determine their different interaction modes.

**Table 2:** Different polar and nonpolar contributions to the binding free energies in MTX-IKCa and MTX-Kv1.2 complexes

Complex	*ΔE*_elec_	*ΔE*_vdW_	*ΔE*_inter_	*ΔΔG*_GB_	*ΔΔG*_SA_	ΔG*_binding_
MTX-IK_Ca_	-118.21	-78.65	-196.86	163.92	-10.59	-43.53
MTX-Kv1.2	-1577.51	-74.06	-1651.57	1616.66	-10.55	-45.46

Binding free energies (kcal/mol) were calculated using the MM-GBSA program in Amber-8.

## Conclusions

Through combined computational methods, including ZDOCK, clustering analysis, and MDS, a reasonably stable MTX-IK_Ca _complex structure was obtained. Further study of this structure showed that in spite of sharing similar pharmacological profiles toward both IK_Ca _and Kv1.2 channels, MTX associated with the IK_Ca _channel in a quite different mode compared to that of MTX interacting with the Kv1.2 channel. In the bound complex, MTX assumed the β-sheet domain as the interaction surface with the Lys23 occluding the pore of the IK_Ca _channel in a manner similar to its interaction with the Kv1.2 channel. However, the conformation of another key residue Tyr32, which was the key to the stability of the complex structure, differed greatly when MTX recognized the IK_Ca _channel, compared to the process with the Kv1.2 channel. It continued lying on the linker connecting the selectivity filter and the S6 helix of the IK_Ca _channel, forming strong polar and nonpolar interactions with residues on the pore region of the channel. In addition, the Lys7 of MTX is possibly involved in the toxin-channel interface reorganization process; however, it does not contact any residues of the IK_Ca _channel directly in the final conformation. This is in contrast with the fact that the Lys7 of MTX formed strong polar interactions with the Asp373 at the pore region of the Kv1.2 channel. In addition, electrostatic and vdW interactions contributed equally to the binding of MTX with IK_Ca_, whereas the MTX-Kv1.2 association featured dominant electrostatic contribution. Such conformational and energetic differences in recognition could be well explained by the different functional roles of the channel vestibules. The longer, neutral-charged IK_Ca _channel turret played an important role in stabilizing the final IK_Ca_-MTX complex, with four residues--Gln229, Ala230, Ala233, and Thr234 forming polar and non-polar interactions with MTX. On the contrary, the shorter Kv1.2-channel turret is highly negatively charged and is barely involved in recognizing MTX. In all, the differences in the binding mechanisms of MTX toward the IK_Ca _and Kv1.2 channels unveiled in this study could offer a better understanding of the physicochemical properties and conformational distinctness of the two channels and thus give a hint for designing MTX-derived inhibitors to discriminate between these two channels.

## Methods

### Atomic Coordinates and Molecular Docking

The atomic coordinates of MTX (PDB code: 1TXM) were downloaded from the PDB [28]. The previous segment-assembly homology model was applied to obtain the structure of the pore region of the IK_Ca _channel [24]. This model was then subjected to 5-nanosecond (ns) MDS for equilibration.

To improve the docking performance, all 35 conformations of MTX were used to dock with the equilibrated IK_Ca _structure through the ZDOCK program [29], a fast Fourier transform (FFT)-based, initial-stage rigid-body molecular-docking algorithm. Each docking produced 1000 candidate complexes, thus 35000 candidate MTX-IK_Ca _complexes were obtained and used for the clustering analysis. According to the orientation of the MTX β-sheet domain, the 35000 complexes were then divided into four main binding modes. Clustering analysis and experimental data-based screening [17,18] were then carried out on all the complexes to select the possible hits from all modes. Candidates from each binding mode were then subjected to a 500-step energy minimization using the Sander module of the Amber-8 suit of programs [30]. By calculating the ligand-receptor binding energies with the ANAL program of Amber-8, appropriate candidate complexes were identified for further MDS study.

### MDS study

All the simulations in this work were carried out using the Amber-8 program [30] on a 64-CPU Dawning TC4000L cluster (Beijing, China). The generalized Born model [31], which has been successfully used to study other toxin-channel interactions [22,24,25,32,33], was applied in this study.

All the candidate complexes selected by the screening process went through 400-picosecond (ps) equilibration and 500-ps unrestrained simulations to introduce more flexibility. The equilibration steps were taken by gradually reducing the force constant--from 5.0 (kcal/mol)/Å^2 ^for restraining all the heavy atoms, to 0.02 (kcal/mol)/Å^2 ^for heavy atoms of the backbone only. The temperature was set at 300 K, with a cutoff distance of 12 Å. For the most reasonably stable complex selected after a 500-ps unrestrained simulation, an additional 10-ns unrestrained simulation was conducted to introduce enough flexibility and to probe into the interaction details. Throughout all the energy minimization and simulation processes, the ff99 force field (Parm 99) [34] was applied.

During the simulation, the membrane around the channel has not been taken into account. It is because that the scorpion peptide binds to the extracellular part of the channel according to mutagenesis studies and solid-state NMR results [6,35-37], where the interaction is hardly affected by the membrane and the transmenbrane segment of channel. Other study groups have also used the same membrane-ignoring measures in molecular simulation studies of toxin-channel interactions [22-25,32,33,38-40]. However, the importance of the membrane in the functioning of channels has been increasingly recognized. A transmembrane protein system could be more reliable if the role of the membrane were taken into account.

### Calculation of Free energy of Binding by the Molecular mechanics--Generalized Born Surface Area method

In the molecular mechanics--generalized born surface area (MM-GBSA) method of AMBER-8 [30], the free energy of binding of the reaction A + B → AB is calculated using the following thermodynamic cycle:

(1) $$\begin{array}{c} \Delta G_{\text{binding}}=\Delta G_{\text{gas}}-\Delta G_{\text{Solv}}^{\text{A}}-\Delta G_{\text{solv}}^{\text{B}}+\Delta G_{\text{solv}}^{\text{AB}}\\ =\Delta H_{\text{gas}}-T\Delta S-\Delta G_{\text{GBSA}}^{\text{A}}-\Delta G_{\text{GBSA}}^{\text{B}}+\Delta G_{\text{GBSA}}^{\text{AB}}\\ =\Delta H_{\text{gas}}-T\Delta S+\Delta \Delta G_{\text{GB}}+\Delta \Delta G_{\text{SA}} \end{array}$$

(2) $$\Delta H_{\text{gas}}\approx \Delta E_{\text{gas}}=\Delta E_{\text{intra}}+\Delta E_{\text{elec}}+\Delta E_{\text{vdW}}$$

(3) $$\Delta \Delta G_{\text{GB}}=\Delta G_{\text{GB}}^{\text{AB}}-(\Delta G_{\text{GB}}^{\text{A}}+\Delta G_{\text{GB}}^{\text{B}})$$

(4) $$\Delta \Delta G_{\text{SA}}=\Delta G_{\text{SA}}^{\text{AB}}-(\Delta G_{\text{SA}}^{\text{A}}+\Delta G_{\text{SA}}^{\text{B}})$$

where *T *is the temperature, *S *is the solute entropy, Δ*G*_gas _is the interaction energy between A and B in the gaseous phase, and $\Delta G_{\text{solv}}^{\text{A}}$, $\Delta G_{\text{solv}}^{\text{B}}$, and $\Delta G_{\text{solv}}^{\text{AB}}$ are the solvation free energies of A, B, and AB, which are estimated using the GBSA method [30]. That is, $\Delta G_{\text{solv}}^{\text{AB}}=\Delta \Delta G_{\text{GBSA}}^{\text{AB}}+\Delta G_{\text{GB}}^{\text{AB}}+\Delta G_{\text{SA}}^{\text{AB}}$, and so forth. Δ*G*_GB _and Δ*G*_SA _are the electrostatic and nonpolar terms, respectively. Δ*E*_bond_, Δ*E*_angle_, and Δ*E*_torsion _are contributions to the intramolecular energy Δ*E*_intra _of the complex. *E*_vdW _is vdW interaction energy. Because of the constant contribution of -*T*Δ*S *for each docked complex, we quote ΔG*_binding _for Δ*G*_binding _+ *T*Δ*S *in the discussion. To verify the quality and validity of the resulting MTX-IK_Ca _complexes, the relative free energy of binding, ΔG*_binding_, was calculated using MM-GBSA for postprocessing-collected snapshots from the MD trajectories. In this work, 30 snapshots from the last 30-ps MDS were used for analysis of the free energy of binding.

## Authors' contributions

HY conceived of the study, analyzed the data and drafted the manuscript. SQ performed the docking analysis, carried out the molecular simulation studies and analyzed the data. YLW and WXL participated in its design and coordination, and helped to draft the manuscript. BSW supervised the study design, coordination and edited the manuscript. All authors read and approved the final manuscript.

## Supplementary Material

Additional file 1 **The interaction details between the D chain of IK_Ca _channel and MTX**. (A) Gln229 on the channel turret interacts with Gly18 and Pro20 of MTX. (B) Ala230 on the channel turret interacts with Pro20 of MTX. (C) Ala233 on the channel turret interacts with Thr17 of MTX. (D) Thr234 on the channel turret interacts with Tyr32 and Gly33 of MTX. Click here for file
