# Supplementary figures and images for: Differential molecular information of maurotoxin peptide recognizing IKCa and Kv1.2 channels explored by computational simulation
Source: BMC Struct Biol. 2011 Jan 25;11:3. doi: 10.1186/1472-6807-11-3 (PMC3041654; doi:10.1186/1472-6807-11-3)

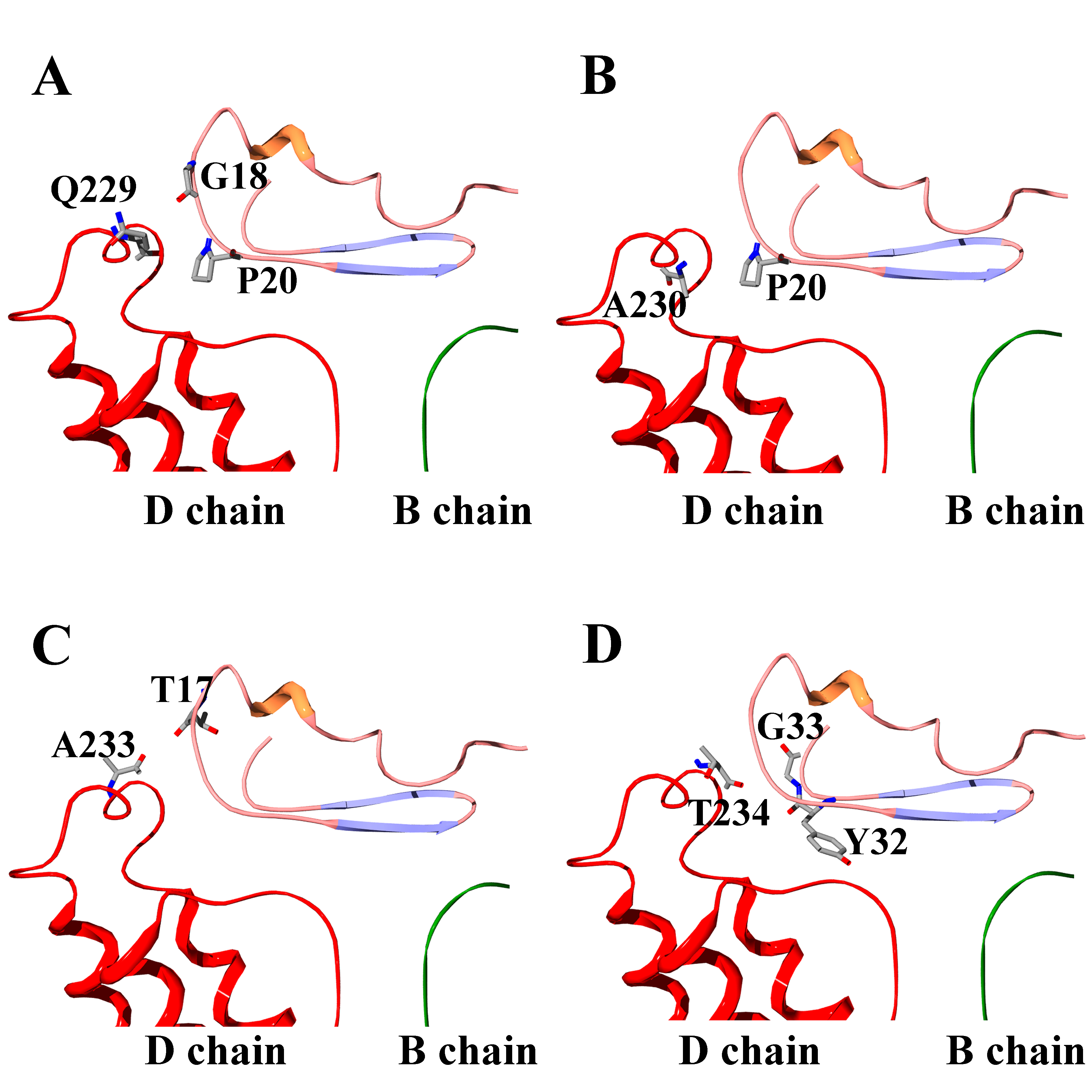

Supplement: Additional file 1 — The interaction details between the D chain of IKCa channel and MTX. (A) Gln229 on the channel turret interacts with Gly18 and Pro20 of MTX. (B) Ala230 on the channel turret interacts with Pro20 of MTX. (C) Ala233 on the channel turret interacts with Thr17 of MTX. (D) Thr234 on the channel turret interacts with Tyr32 and Gly33 of MTX. [file 1472-6807-11-3-S1.JPEG]
